# Nutrient enrichment increases virulence in an opportunistic environmental pathogen, with greater effect at low bacterial doses

**DOI:** 10.1093/femsec/fiae013

**Published:** 2024-02-01

**Authors:** Katja Pulkkinen, Jouni Taskinen

**Affiliations:** Department of Biological and Environmental Science, P.O. Box 35 (Survontie 9), University of Jyväskylä, Jyväskylä, Finland; Department of Biological and Environmental Science, P.O. Box 35 (Survontie 9), University of Jyväskylä, Jyväskylä, Finland

**Keywords:** aquatic environment, bacterium, eutrophication, fish disease, *Flavobacterium columnare*, nutrient

## Abstract

Eutrophication of aquatic ecosystems is associated with an increased risk of pathogen infection via increased pathogen growth and host exposure via increased pathogen doses. Here, we studied the effect of nutrients on the virulence of an opportunistic bacterial pathogen of fish, *Flavobacterium columnare*, in challenge experiments with rainbow trout fingerlings. We hypothesized that removing all nutrients by washing the bacteria would reduce virulence as compared to unwashed bacteria, but adding nutrients to the tank water would increase the virulence of the bacterium. Nutrient addition and increase in bacterial dose increased virulence for both unwashed and washed bacteria. For unwashed bacteria, the addition of nutrients reduced the survival probability of fish challenged with low bacterial doses more than for fish challenged with higher bacterial doses, suggesting activation of bacterial virulence factors. Washing and centrifugation reduced viable bacterial counts, and the addition of washed bacteria alone did not lead to fish mortality. However, a small addition of nutrient medium, 0.05% of the total water volume, added separately to the fish container, restored the virulence of the washed bacteria. Our results show that human-induced eutrophication could trigger epidemics of aquatic pathogens at the limits of their survival and affect their ecology and evolution by altering the dynamics between strains that differ in their growth characteristics.

## Teaser

Eutrophication of aquatic ecosystems can increase the risk of pathogen infection and alter the dynamics between strains that differ in their growth characteristics.

## Introduction

Eutrophication of aquatic ecosystems is one of the most serious environmental problems, leading to many negative effects such as algal blooms, oxygen depletion, fish kills, and loss of biodiversity (Smith and Schindler 2009). One of the main concerns associated with anthropogenic nutrient enrichment is the increased risk of infection (McKenzie and Townsend 2007, Johnson et al. 2010, Aalto et al. 2015). Nutrient enrichment has been associated with increased infections in various aquatic organisms, such as fungal infections in corals (Voss and Richardson 2006), helminth infections in amphibians (Johnson et al. 2007), and myxozoan infections in fish (Bailey et al. 2018, Lauringson et al. 2021).

Many pathogens that cause disease epidemics in aquatic organisms can survive and replicate in the aquatic environment without the host (Arias et al. 2012, Sundberg et al. 2014, Ben Hamed et al. 2018, Zhang et al. 2020). In natural waters without nutrient or organic pollution, the nutrient levels are usually low (Wetzel 2001), and numbers of microbial pathogens are so low that successful host invasions are unlikely. Many aquatic microbes are adapted to persist in low-nutrient water while retaining the ability to reproduce under nutrient replete conditions (Elser et al. 1995, Cotner et al. 2010).

The eutrophication of aquatic ecosystems or the accumulation of organic nutrients in the water can create conditions that favor disease outbreaks in aquatic organisms (Smith and Schindler 2009, Kinnula et al. 2017). Increased nutrients can promote pathogen growth and consequently increase the risk of infection by increasing the infectious pathogen dose encountered by the host (Smith and Schindler 2009, Wedekind et al. 2010, Kinnula et al. 2017). Increased organic and inorganic nutrient enrichment of the environment outside the host may affect the growth and characteristics of environmentally growing opportunistic pathogens, including their virulence (Brown et al. 2012). Furthermore, if the resources available in the outside-host environment are nutritionally close to the host tissues, the pathogen could become preadapted for host exploitation (Brown et al. 2012, Ketola et al. 2016), possibly via activation of virulence factors (Penttinen et al. 2016, Kinnula et al. 2017). Understanding the effects of nutrient enrichment on the dynamics and evolution of aquatic pathogens, both in natural waters and in aquaculture, is important for predicting and mitigating their impact on aquatic ecosystems and food production.

*Flavobacterium columnare* is an aquatic pathogen that infects a wide range of freshwater fish, including important aquaculture species such as channel catfish and salmonids (Declercq et al. 2013). The pathogen attacks the gills, skin, and fins of fish, causing tissue damage and necrosis, that is ultimately fatal to the host. Infection with highly virulent strains can kill the host before visible signs of disease appear. The pathogen can multiply in live and dead host fish, and other organic decaying material in the water (Kunttu et al. 2009, 2012). *Flavobacterium columnare* can be isolated from lake water (Kunttu et al. 2012), and has been shown to survive in lake water and distilled water for long periods in the laboratory (Arias et al. 2012, Sundberg et al. 2014). In the natural environment, the bacterium is likely to encounter more oligotrophic conditions than in the aquaculture facilities, where water can contain high concentrations of nutrients from fish feed and excreta (Naylor et al. 1999). Increased nutrient concentrations in the growth medium (Penttinen et al. 2016) or in tank water (Kinnula et al. 2017) have been shown to increase the virulence of *F. columnare* in challenge tests with rainbow trout (*Oncorhynchus mykiss*) and zebrafish (*Danio rerio*). Organic nutrients in the water of fish farms could therefore promote disease epidemics caused by *F. columnare* on farms.

Here, we studied the effect of nutrients in the environment outside the host on the virulence of *F. columnare* in fish challenge experiments using five different doses of bacteria. We compared the virulence of fresh, unwashed bacteria with bacteria that had been centrifuged, washed, and diluted in distilled water to remove growth media. We then examined how the virulence of these bacteria responded to a small addition of growth media (0.05%), added separately to the fish tank. We hypothesized that removing all nutrients by washing would reduce virulence but adding nutrients to the tank water would increase virulence of the bacterium to rainbow trout fingerlings. We show that organic nutrients in the water increase virulence, and especially so at low bacterial doses, and in washed and possibly damaged bacterial cells. Our results suggest that environmental nutrient enrichment may be more important than previously thought in maintaining and initiating disease epidemics in aquatic organisms.

## Materials and methods

*Flavobacterium columnare* strain B067 stored frozen in −80°C with 10% fetal calf serum and 10% glycerol was resuspended in modified Shieh medium (Song et al. 1988), further modified by excluding yeast extract and replacing peptone with casamino acids, and cultured overnight at room temperature. The overnight culture was subcultured for a further 18 h by adding 2 ml of the overnight culture into 20 ml of fresh medium. After combining all subcultures, bacterial culture was divided into five 50 ml tubes. Four of the tubes were centrifuged (3500 *g*, 4°C, and 12 min), the liquid supernatant was removed, and the pellets were diluted back to 50 ml of distilled sterile water. This procedure was repeated twice, after which the pellets from all four tubes were combined and diluted in 21 ml of distilled sterile water. One remaining unwashed 50 ml tube of the bacterial culture was used as such in the fish challenge experiments (see below). Both the washed and unwashed bacterial solutions were diluted at 1:2, 1:5, 1:10, and 1:20 with distilled sterile water. Including the undiluted cultures, there were 10 separate bacterial treatments in total to be used in fish challenges. Viable counts of undiluted solution and solutions diluted to 1:2 and 1:20 were then determined from plate counts of serial dilutions (10^0^–10^−7^) on Shieh agar plates. The viable counts for the 1:5 and 1:10 dilutions were calculated based on counts from the more concentrated cultures assuming that the change in bacterial numbers was proportional to the dilution of the liquid.

The fish challenge experiments tested the effect of washing the bacteria, bacterial dose, and nutrient addition on bacterial virulence. Rainbow trout (*O. mykiss*) fingerlings were obtained from a fish farm using groundwater and were therefore disease-free. The fish were maintained in the laboratory in aerated well water with a 12:12 L:D cycle at 17°C and fed with commercial pellets. Prior to the experiment, the water temperature in the fish holding tanks was gradually increased from 16.4 to 25.5°C over 8 days to acclimate the fish to higher temperature. Higher temperature was used to ensure rapid onset of the disease to meet the humane endpoints of the experiment. Rainbow trout fingerlings (0.8 ± 0.3 g) were individually transferred to 500 ml plastic containers filled with borehole water (25.5°C) aerated immediately before the experiment. Fish were challenged using the continuous exposure method (Kinnula et al. 2015). Briefly, 500 µl of each bacterial dose (1:0, 1:2, 1:5, 1:10, and 1:20) of either washed or unwashed bacteria was added to the containers, for a total of 10 treatments. A total of 10 additional treatments (each of the five doses for both washed and unwashed bacteria) received an addition of 500 µl of fresh Shieh medium. Specifically, the bacteria were not mixed with the nutrient medium prior to addition to the fish containers, but the medium was added separately after the addition of the bacteria on the opposite side of the container. There was no aeration in the containers, so bacteria and nutrients were mixed to the water only due to fish movements. After exposure, the fish containers were placed in random order on the shelves in a temperature-controlled room at 25.5°C. Each bacterial dose x nutrient treatment (without or with nutrient addition) combination was replicated ten times, except for the treatment with unwashed bacteria and nutrient addition with the lowest dose, which had only nine fish, resulting in 199 challenged fish. In addition, 30 fish received a 500 µl addition of unwashed Shieh medium, and 20 fish received a 500 µl addition of sterile water. A total of 249 fish were used in the experiment.

Fish were monitored hourly for signs of disease and those showing signs of disease (moribund, unbalanced swimming, and unresponsive to external stimuli) were euthanized with Tricaine mesylate solution (MS-222, Sigma) and killed by decapitation with scissors. Bacterial samples from fish gills were plated on Shieh agar plates containing tobramycin (Decostere et al. 1997) to verify infection with *F. columnare*. Fish weights were recorded. The experiment was terminated after 30 h, after which all surviving fish were killed with an overdose of MS-222. The experiment was conducted according to the Finnish Act on the Use of Animals for Experimental Purposes. The license was granted by the National Animal Experiment Board at the Regional State Administrative Agency for Southern Finland (permission number ESAVI/10184/04.10.07/2014).

### Data analyses

For fish challenges with the unwashed bacteria, the fish survival was analyzed with Cox survival analysis, with nutrient addition as categorical, and bacterial dose (CFU ml^−1^, log-transformed, with mean standardized to zero) and fish weight as continuous variables. Model selection was based on the Akaike information criteria starting from the full model including a three-way interaction and all 2-way interactions. The proportional hazards assumption was checked with Schoenfeld residuals test (*P* > .05 for all tested factors) (In and Lee 2019). For fish challenges with the washed bacteria, the lack of mortality in treatment without added nutrients led to problems with model convergence in cox-regression, hence Kaplan–Meier log-rank test was used for comparing the two nutrient levels. Statistical analyses were performed using R-studio version 2022.12.0.353 (RStudio Team 2020), with package “survival” (Therneau 2023).

Due to the statistical limitations in performing survival analyses, mean fish longevity was further analyzed. For each bacterial dose and nutrient treatment, one-tailed one-sample *t*-tests were used to test whether the mean survival time was less than the maximum survival time in the experiment (30 h). Bonferroni-correction was applied to adjust the probability of increased risk of type I error associated to multiple testing. In addition, the percentage of survived fish was calculated for each bacterial dose and nutrient treatment, but as most of these were either 0% or 100%, they were not statistically analyzed, but are shown for comparison.

## Results

Based on plate counts of unwashed and washed bacteria, washing resulted in ~1000 times lower bacterial doses administered to fish. The doses at dilutions 1:0, 1:2, 1:5, 1:10, and 1:20 for the unwashed bacteria were 3.5 × 10^4^, 1.8 × 10^4^, 7.1 × 10^3^, 3.6 × 10^3^, and 1.8 × 10^3^ CFU ml^−1^, while those for the washed bacteria were 4.2 × 10^1^, 2.1 × 10^1^, 8 × 10^0^, 4 × 10^0^, and 2 × 10^0^ CFU ml^−1^. Due to this large difference in CFU ranges for the washed and unwashed bacteria, the data were analyzed separately for fish challenged with unwashed and washed bacteria. Of the 20 control fish with the addition of 500 µl of sterile water, one died at time point 19 h, and of the 30 control fish with the addition of 500 µl of fresh Shieh growth medium, one died at time point 27 h. However, *F. columnare* was not recovered from the bacterial gill samples of these two fish.

**Figure 1.**
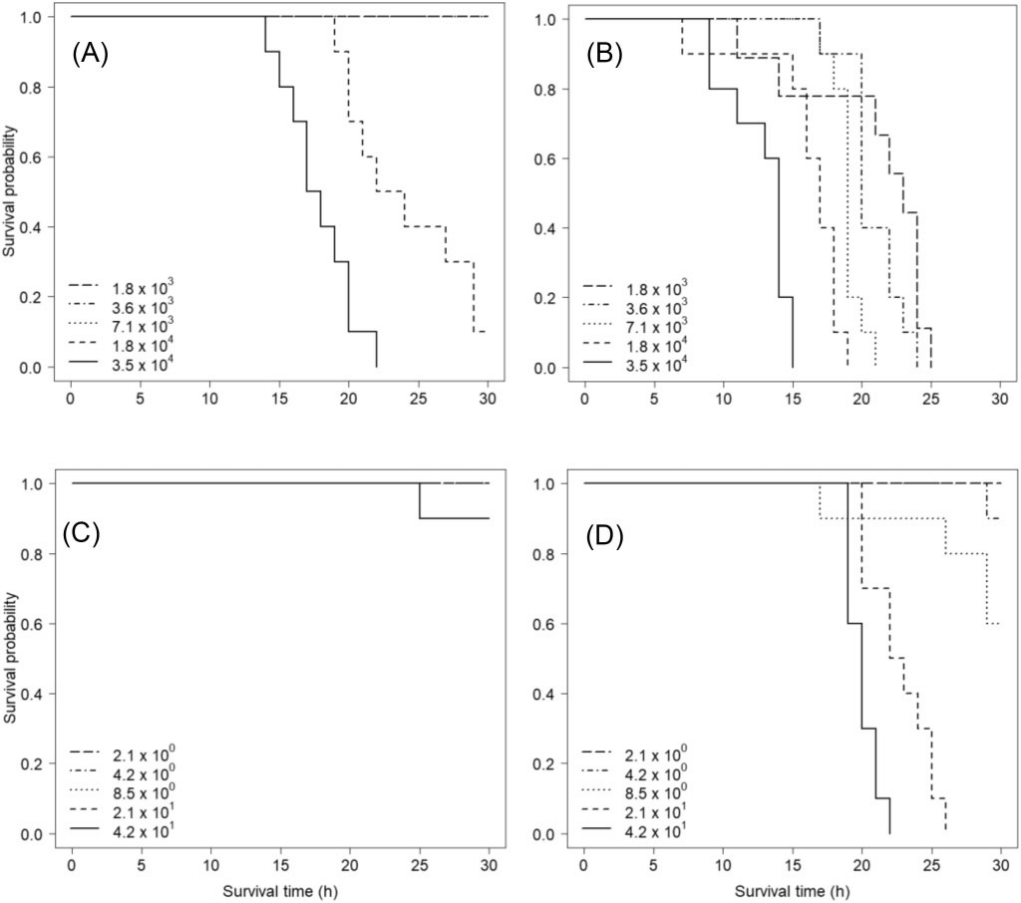
Kaplan–Meier survival probabilities for rainbow trout (*O. mykiss*) fingerlings challenged with *F. columnare*. Fish were challenged with five different doses (CFU ml^−1^) of (A) unwashed bacteria without nutrients, (B) unwashed bacteria with nutrient addition, (C) washed bacteria without nutrients, and (D) washed bacteria with nutrient addition. Note that the doses differ between unwashed and washed bacteria.

The best model explaining fish survival in challenge experiment with unwashed bacteria included nutrient addition, bacterial dose, and their interaction. Fish weight had no effect on survival probability. Nutrient addition decreased fish survival probability (Exp(b):604.2; 95% CI: 78–4657; Wald: 6.146, *P* < .001). Survival probability also decreased with increase in bacterial dose (CFU) (Fig. 1A and B; Exp(b):93.4 for log-transformed values; 95% CI: 19–466; Wald: 5.530, *P* < .001). The interaction between nutrient addition and bacterial dose was statistically significant, indicating that when nutrients were added, bacterial dose had less effect on the survival probability (Fig. 1A and B; Exp(b):0.06; 95% CI: 0.01–0.27; Wald: −3.587, *P* < .001), i.e. also the fish challenged with low bacterial doses died.

The addition of nutrients also reduced the survival probability of fish challenged with washed bacteria, (Fig. 1C and D; log rank Χ^2^ = 30.5, df = 1, *P* < .001). When nutrients were not added with the washed bacteria, bacterial dose had no effect on fish survival (Fig. 1C; log rank Χ^2^ = 4, df = 4, *P* = .4), but when nutrients were added, survival probability decreased with increasing bacterial dose (Fig. 1D; log rank Χ^2^ = 65.4, df = 4, *P* < .001).

**Figure 2.**
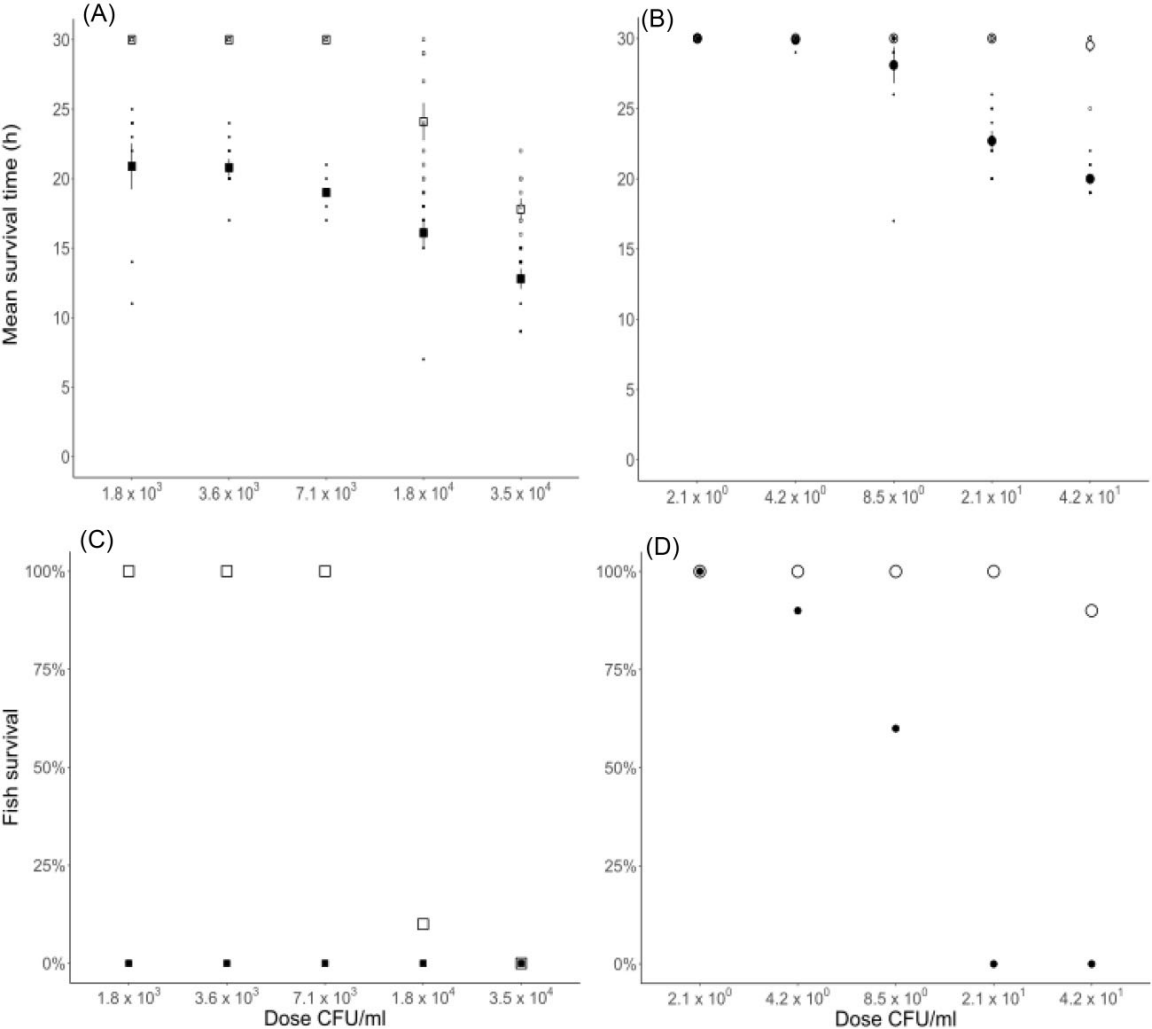
Survival times of individual fish and mean survival time ± SE (h) and survival percentage (%) of rainbow trout (*O. mykiss*) fingerlings challenged with *F. columnare*. Fish were challenged with five different doses (CFU ml^−1^) of (A), (C) unwashed *F. columnare* and (B), (D) washed *F. columnare*, both without (open symbols) or with nutrient addition (filled symbols). Note, that the *x*-axis scales (doses) are different for the unwashed (left panel) and the washed bacteria (right panel).

For fish challenged with unwashed bacteria the mean survival time was not different from 30 h (maximum survival to the end of the experiment) for the three lowest bacterial doses (Fig. 2A; one- sample *t*-tests, one-sided *P* > .05), i.e. all fish survived in these treatments (Fig. 2C). At the two highest bacterial doses, the mean survival time was statistically significantly less than 30 h (Fig. 2A; dose 3.5 × 10^4^ CFU ml^−1^, *t* = −15.5, df = 9, *P* < .001 and dose 1.8 × 10^4^ CFU ml^−1^, *t* = −4.4, df = 9, *P* < .01), and fish survival was less than 10% (Fig. 2C). When nutrients were added with unwashed bacteria, the mean survival time was statistically significantly less than 30 h for all bacterial doses (Fig. 2A; dose 3.5 × 10^4^ CFU ml^−1^, *t* = −23.7, df = 9, *P* < .001; dose 1.8 × 10^4^ CFU ml^−1^, *t* = −12.9, df = 9, *P* < .001; dose 7.1 × 10^3^ CFU ml^−1^  *t* = −33, df = 9, *P* < .001; 3.6 × 10^3^ CFU ml^−1^; *t* = −14.6, df = 9, *P* < .001; 1.8 × 10^3^ CFU ml^−1^; *t* = −5.5, df = 8, *P* < .01). None of the fish challenged with unwashed bacteria and added nutrients survived (Fig. 2C). Nutrient addition, thus decreased fish survival relatively more at the three lowest bacterial doses than at the two highest doses, as at the three lowest bacterial doses no mortality occurred without added nutrients.

The mean survival time of fish challenged with washed bacteria did not differ from 30 h (maximum survival time to the end of the experiment) for any of the five bacterial doses given (Fig 2B; one-sample *t*-tests, one-sided *P* > .05). Mortality occurred only at the highest dose (Fig. 2D). For fish challenged with washed bacteria and nutrient addition, the mean survival time was not different from 30 h for the three lowest bacterial doses (one-sample *t*-tests, one-sided *P* > .05), but was statistically significantly lower than 30 h for the two highest doses (dose 4.2 × 10^1^ CFU ml^−1^, *t* = −10.2, df = 9, *P* < .001 and dose 2.1 × 10^1^ CFU ml^−1^, *t* = −30, df = 9, *P* < .001). When nutrients were added to the washed bacteria, mortality occurred at all but the lowest bacterial dose (Fig. 2D).

## Discussion

Here, we showed that a small addition of nutrient medium (0.05% of water volume), added separately to the fish container, restored the virulence to washed *F. columnare* bacteria, whereas without nutrient addition the washed bacteria were unable to infect fish. In addition, when challenged with unwashed bacteria, the addition of nutrients reduced fish survival probability and survival time relatively more at the lowest doses of *F. columnare* than at the higher bacterial doses. In natural, unpolluted water environments, bacterial abundances are generally low (Wetzel 2001) and pathogens represent only a fraction of the bacteria. Hence, the doses of microbial pathogens encountered by the hosts in natural waters are low as compared to the pathogen doses required for confirmed infections under experimental conditions (Kinnula et al. 2015, Sundberg et al. 2016). The current results suggest that when pathogenic bacteria are in poor condition or when their numbers are low in natural unpolluted water environment, presence of organic nutrients in water may have a critical importance in restoring the virulence of the pathogenic bacteria. The results support previous findings on the effect of human-induced environmental nutrient enrichment in maintaining and triggering disease epidemics in aquatic organisms (Wedekind et al. 2010, Kinnula et al. 2017, Pulkkinen et al. 2018, Zhang et al. 2020).

Our results from the challenges with the different doses of unwashed bacteria confirmed the findings of previous studies on the dose-dependent infection probability of *F. columnare* (Kinnula et al. 2015, 2017). Previous studies have shown that addition of extra nutrients in the growth medium or aquarium water increases the virulence of aquatic bacterial pathogens (Wedekind et al. 2010, Penttinen et al. 2016, Kinnula et al. 2017). Increased nutrient availability is likely to increase bacterial replication and hence the pathogen dose contacted by the host, thereby increasing the likelihood of infection. A previous study demonstrating increased virulence associated with nutrient addition used 0.64%–1.28% nutrient addition with fresh, unwashed cultures that may also contain nutrients from the original growth medium (Kinnula et al. 2015). In our experiment, the nutrient addition (0.05%) was an order of magnitude lower, and it was added separately to the other side of the fish container, so that the added bacteria were not in direct contact with the nutrient broth when dispersing into the water in the container solely due to fish movements. Therefore, it is unlikely that the increased virulence due to nutrient addition in our experiment was achieved solely via increased growth of the bacteria.

In current experiments, fish survival was relatively lower at the low doses than at the high doses of the unwashed bacteria when nutrients were added. The study by Kinnula et al. (2017) did not find an interaction between bacterial dose and nutrients but did find increased virulence in a low virulent strain in the highest nutrient treatment. Our results support the suggestion that increased virulence in the presence of outside-host nutrients is achieved in part through activation of virulence factors (Kinnula et al. 2015, Penttinen et al. 2016). In *F. columnare*, increased nutrient levels have been shown to activate the putative virulence factors chondroitinase (cslA) and collagenase (Penttinen et al. 2016).

Based on plate counts, there was an extensive loss of cell viability after washing, centrifugation, and subsequent dilution in distilled water in our experiment. Washing and subsequent centrifugation are standard laboratory procedures used to remove external contaminants from cells under investigation (Peterson et al. 2012). High-speed centrifugation (15 000 × *g*) is known to damage Gram-negative bacterial cells due to the shear forces caused by cell collisions (Pembrey et al. 1999, Peterson et al. 2012). On the other hand, the centrifugation speed in our experiment was below the level at which significant cell damage has been found (5000 × *g*) and which is used as a reference method in experiments (Pembrey et al. 1999). It is possible that the long rod-like cell morphology (0.3–0.5 µm wide × 3–10 µm long) of *F. columnare* cells makes them more sensitive to damage during centrifugation than the coccoid cells or short rods previously tested (Pembrey et al. 1999, Declercq et al. 2013).

Dilution of bacterial cells to distilled water alone may not have been detrimental to *F. columnare* cells, as *F. columnare* can remain viable in distilled water for months (Arias et al. 2012, Sundberg et al. 2014). Long-term survival in distilled water has been suggested to be based on the recycling of nutrients from dead and decaying cells (Kunttu et al. 2009), but cell division of starved cells under the microscope has not been demonstrated (Arias et al. 2012). *Flavobacterium columnare* cells appear to remain culturable even under prolonged starvation (Arias et al. 2012), unlike some other aquatic pathogenic bacteria such as *Aeromonas hydrophila* (Rahman et al. 2001), *F. psychrophilum* (Madetoja et al. 2003), or *Francisella noatunensis* (Duodu and Colquhoun 2010), which enter a nonculturable but viable state. Instead, *F. columnare* cells undergo a change in cell morphology from the long rod shape to a coiled resting form (Arias et al. 2012). Diversification of colony morphology detected during prolonged starvation also suggests that cell morphology undergoes changes during starvation (Sundberg et al. 2014).

The loss of virulence in fish challenge experiments after washing and centrifugation was probably due to damage to cell surface molecules, which are important virulence factors for *F. columnare* (Kunttu et al. 2021). The importance of surface molecules for virulence in *F. columnare* has been demonstrated, for example, in experimental infections of *F. columnare* cells with phages, which led to a change in colony morphotype with a concomitant loss of virulence (Laanto et al. 2012, Kunttu et al. 2021). Surface molecules affect bacterial properties such as adhesion, colony morphotype, and biofilm formation, all of which may contribute to fish epithelial colonization and hence bacterial virulence (Decostere et al. 1997, Kunttu et al. 2011, Olivares-Fuster et al. 2011, Lange et al. 2017). In addition to surface molecules, bacterial virulence is affected by proteins and polymeric substances (EPS) that bacteria excrete outside the cells (Newton et al. 1997, Laanto et al. 2014, Lange et al. 2017, Zhang et al. 2017, Mekasha and Linke 2021). The EPSs are excreted via specialized secretion systems, of which T9SS has been associated with several virulence factors in *F. columnare* (Kunttu et al. 2021, Mekasha and Linke 2021), such as chondroitin AC lyase and metalloproteases that degrade connective and muscle tissue (Newton et al. 1997). Centrifugation and washing may have removed these substances or altered their expression, and hence the virulence properties of the bacterial cells.

Interestingly, the presence of fish and the potential nutrients in fish excreta in the water alone were not sufficient to induce infection when the washed *F. columnare* cells were added to fish containers without added nutrients. Fish mucus can support the growth of *F. columnare* (Shoemaker et al. 2018) and *F. columnare* biofilms (Lange et al. 2020) and increase the expression of some extracellular proteins (Staroscik and Nelson 2008, Almeida et al. 2019), all of which could aid in the infection of fish hosts. In addition, the nutrients in fish excreta could support bacterial growth.

According to the plate count, the doses of the unwashed bacteria were three orders of magnitude higher than doses of the washed bacteria, but when nutrients were added, the survival times of the fish challenged with the two highest doses (4 × 10^1^ and 2 × 10^1^ CFU ml^−1^) of the washed bacteria were not much lower (20–25 h) than that of the unwashed bacteria (15–25 h), and none of the fish survived. The plate count may have underestimated the abundance of the washed cells because there was a longer interval between preparation of the washed solution and plating than between washing and challenge of the fish. Another possibility is that washed bacteria formed aggregates, resulting in higher doses added to fish containers than detected by plate counting. Aggregation of *F. columnare* cells has been detected after Ca_2_Cl_2_ addition to TYES medium (Evenhuis et al. 2021) or after mucin addition to Shieh medium (Almeida et al. 2019), as well as in our experience after dilution of the Shieh nutrient medium. However, the dilution series of washed bacteria with nutrient addition also appeared to function in a dose-dependent manner, based on reduction in mean host longevity and proportion of fish surviving with the increase in bacterial dose.

In conclusion, our results suggest that nutrients in the outside host environment of the bacteria may play a crucial role in maintaining and initiating disease epidemics. In *F. columnare*, there is a positive association with growth and virulence (Pulkkinen et al. 2010, 2018, Kinnula et al. 2017), as well as a dose-dependent increase in host infection risk (Kinnula et al. 2017), suggesting that the virulent strains generally win in the competition for host invasion. Here, we have shown that organic nutrients can restore virulence to bacterial cells that are on the verge of losing viability in water, and that nutrients increase virulence relatively more at the low bacterial doses than at the high doses. In the natural, less eutrophic waters, some pathogenic bacteria may persist in low numbers at the limits of their viability. Anthropogenic nutrient enrichment of the environment could promote the growth of these slow-growing pathogens to reach the doses required for host invasion and increase their contribution to epidemics, thus changing the dynamics between pathogen strains. Nutrient enrichment may therefore have a greater impact on the ecology and evolution of pathogens in aquatic organisms than previously thought, both in natural waters and in aquaculture.
